# A commentary on theory of mind

**DOI:** 10.3389/fpsyg.2015.01560

**Published:** 2015-10-14

**Authors:** Marilyn Shatz

**Affiliations:** ^1^Psychology and Linguistics, University of MichiganAnn Arbor, MI, USA; ^2^Psychology, University of North Carolina WilmingtonWilmington, NC, USA

**Keywords:** mind-reading, behavior-reading, children, animals, modularity of mind

## The child as philosopher

“You don't know what I'm thinking,” my 3-year-old granddaughter called to me from her car seat. Her out of-the-blue, metacognitive comment confirmed that, despite inconsistent performance on false belief tasks, young children reveal sophisticated mindreading abilities in their spontaneous talk (Shatz, 1994). At the least, she was concerned with knowledge in another's mind. But her statement suggested more: With no prior conversational context, and serving no communicative or behavioral purpose, it seemed to be in the tradition of philosophy of mind. What kind of theory of mind (TOM) lay behind it?

## Possible theories of mind

As scientists, we must consider the most economical, reasonable explanations for behavior. Possibly, a minimal TOM, e.g., behavior-reading as clues to intention (Butterfill and Apperly, 2013), could have accounted for her comment. Because I was driving and directing my gaze elsewhere while she sat quietly, she probably knew that I lacked perceptual information about her. (Behavior-reading handles well the findings on animal “mindreading,” Lurz, 2011). However, comments about mindreading from competent language users like her may exemplify more, namely, thinking about mind. Possibly she held “the doctrine of opacity of others' minds,” the belief that it is near impossible to know what another is thinking (Robbins and Rumsey, 2008).

In numerous Melanesian cultures, talk about others' thoughts is inappropriate, but evidence is lacking that mindreading does not happen (see Keane, 2008). Thus, the universality of mindreading among human adults has not been seriously challenged. Indeed, discoveries with pre-linguistic infants and animals have encouraged the view that, while possibly necessary for mature mindreading, language is very likely subsequent to early TOM skills (e.g., Malle, 2002). Apparently, children in Melanesian cultures still read others' minds, but they must learn not to talk about them (Schieffelin, 2008).

In Western cultures, social experience and family talk about mental states fosters TOM development (see Antonietti et al., 2006). As the younger sibling in an upper-middle class American family, my granddaughter very likely heard much talk about mental states and so would have been unrestrained in talking about our minds. Still, one cannot know for sure whether she held a TOM based on behavioral clues or on impossible access.

To explain improving performance with age, TOM theorists have proposed various mechanisms such as a mindreading “module” with performance constraints, growing representational ability, or social experience and language skill. Nichols and Stich (2003) even proposed a multi-component theory drawing on previous accounts. These authors' efforts, as well as later ones, show that no single mechanism explains all the findings on different-aged humans and animals. The answer to the question of who can read minds, when and how, is not simple.

## What is mindreading?

At the least, mindreading entails entity A assessing an internal mental state of entity B that is not accessible from direct perception. Hence, inference on the part of A is necessary. (See Premack and Woodruff, 1979; They coined the term, TOM.) Being a good reader of others' behaviors apparently is insufficient for “full-blown” TOM (Butterfill and Apperly, 2013). Mature mindreading is not constrained to a single (e.g., competitive) circumstance. To account for mindreading-like behaviors in animals and pre-linguistic infants while not granting them mature TOMs, researchers have proposed various minimal or two-system theories (e.g., Apperly and Butterfill, 2009; Ruffman, 2014). Already their proposals have garnered a variety of criticisms (e.g., Carruthers, 2014; Scott, 2014).

So, years after my granddaughter's remark, and after studying many recent arguments, I cannot definitively answer the question of what kind of theory she had. Lurz et al. (2014) propose “optimistic agnosticism” to address whether animals behavior-read or mind-read, but I am not as optimistic as they that further experimentation will establish the truth for either animals or young children. Lurz et al. say that the evidence already favors an innate module, even in animals, as the basis for TOM. The notion of modularity comes from Fodor's (1983) proposal of it as the mind's organizing principle. His work follows from the idea of an innate human language capacity (Chomsky, 1965).

The language and TOM modules are similar in that both have some innate bases or other, and both require environmental input to achieve mature status, that is, “full-blown” TOM and specific language competence. Nonetheless, the modules and their developmental constraints are critically different. The innate language module constrains syntax, allowing humans to use limited data to develop a specific syntactic system. Thus, the syntax module accounts for various features of a language being packaged together so that when a crucial piece of data is encountered, those multiple features can be acquired simultaneously. In contrast, the mindreading module seems to consist of a score of disparate, (albeit possibly innate) abilities functioning together to produce early TOM-like behavior. As these skills grow, so TOM ability grows, bringing success on increasingly difficult tasks. Animal modularity may be different from human modularity altogether, with entirely different constraints (e.g., limited to competitive contexts; Barrett and Kurzban, 2006). More clarity on the nature of TOM modules is needed to decide which, if any, modules share more than an ill-defined label.

The problem of clarity plagues other constructs in TOM proposals as well. For example, the question of what they represent when creatures mind-read is a conundrum because there is no clear definition of representation. Without clear basic constructs, there can be no determinative testing of modular or any other TOM theories.

Only human children can acquire both false-belief understanding and syntactic language. Several researchers have proposed that language is the human ability that can integrate early skills, leading to more advanced ones, (e.g., Spelke, 2003; Shatz, 2007). Or, humans may have a higher-order cognitive capacity that accounts for both language and “full-blown” TOM (see Penn et al., 2008; Shatz, 2008). Such proposals may be agnostic with regard to whether animals' abilities are the evolutionary precursors to adult human strengths, but they are not so with regard to the “pro-discontinuity” position that humans are basically different from animals.

When my 3-year-old granddaughter said, “You don't know what I'm thinking,” my first and lasting impression was that she had marked a crucial difference between us. The grandmother in me credited her with a rather mature TOM. Although, my scientist's head may be more agnostic, it is not at odds with my grandmother's heart. I believe, even after perusing the last decade's work, that young humans have different TOMs from animals. Proof awaits.

### Conflict of interest statement

The author declares that the research was conducted in the absence of any commercial or financial relationships that could be construed as a potential conflict of interest.
